# Exploring Allied Health Models of Care for Children with Developmental Health Concerns, Delays, and Disabilities in Rural and Remote Areas: A Systematic Scoping Review

**DOI:** 10.3390/ijerph21040507

**Published:** 2024-04-19

**Authors:** Georgia Gosse, Saravana Kumar, Helen Banwell, Anna Moran

**Affiliations:** 1 Innovation Implementation and Clinical Translation, Allied Health and Human Performance Unit, University of South Australia, North Tce, Adelaide, SA 5001, Australia; 2 Department of Rural Health, Melbourne Medical School, The University of Melbourne, Graham St Shepparton, Melbourne, VIC 3630, Australia

**Keywords:** rural and remote, allied health, health services, child health, paediatrics, development

## Abstract

Background: Access to appropriate healthcare is essential for children’s healthy development. This is lacking in rural and remote areas, impacting health outcomes. Despite efforts to improve access for these communities, to date, no review has systematically mapped the literature on allied health models of care for children with developmental needs. This scoping review seeks to address this knowledge gap. Methods: Adhering to the PRISMA-ScR and Joanna Briggs Institute guidelines, a systematic search was conducted. A total of 8 databases (from inception to May 2023) and 106 grey literature sources were searched. Two reviewers independently undertook a two-stage screening process. Data were extracted using customised tools and narratively synthesised utilising the Institute of Medicine’s quality domains. This review is registered a priori via Open Science Framework. Results: Twenty-five citations were identified within the literature. Varied models of care were reported from five mostly Western countries. Models of care identified in these areas were classified as screening services, role substitution, consultative services, or online-based services. Positive impacts on quality of healthcare were reported across all quality domains (apart from safety) with the domain of effectiveness being the most commonly reported. Conclusions: Multiple models of care are currently in operation for children with developmental needs in rural and remote areas and appear to improve the quality of care. Due to complexities within, and limitations of, the evidence base, it is unclear if one model of care is superior to another. This review provides a basis for further research to explore why some models may be more effective than others.

## 1. Introduction

Rural and remote areas face significant challenges in health access and service quality globally [1,2,3]. The dispersion of services and resources in these regions, due to low population densities, results in difficulties accessing care and consequently can result in long waitlist times; delayed diagnoses, referrals, and interventions; and poorer continuity of care [2,3,4]. This is of particular concern for vulnerable populations, such as children, the elderly, and those with chronic conditions as they inherently require more specialised services [5,6].

Children residing in remote areas are four times more likely to experience developmental concerns and delays compared to their metropolitan counterparts [7]. Failure to recognise or address developmental delays not only impacts the child but also has broader social, health, and economic consequences, which are compounded in rural areas [8,9]. Recognising the right to support healthy development, the World Health Organization emphasises the need for accessible services, including for children in humanitarian crises, marginalised children, and those with disabilities and developmental delays [10]. Many developmental concerns benefit from intervention from allied health professionals (AHP), particularly those requiring ongoing or more frequent therapy. The challenge with the need for these services then extends beyond children to the AHPs working in rural and remote areas [11]. AHPs play a crucial role in minimising impairments, promoting participation, and reducing long-term vulnerabilities [12]. 

Models of care (MoCs) are a concept used to identify and understand the combination of components of a service or intervention that will have the best chance of ensuring that end-users can access the type of care that they need when they need it, and that care is acceptable, safe, and effective [4]. Various models are used in different contexts with the aim of improving the quality of services delivered to vulnerable groups [4]. In rural areas, a lack of resources, due to myriad contributing factors [4,5,8], makes the development of rural MoCs challenging in nature. Therefore, rural MoCs must be designed with complexity in mind such that they can accommodate rural barriers [13,14]. A large proportion of research examining MoCs in rural settings consequently focuses on the extent to which a particular MoC might address such barriers to enable greater access to care, measuring the impact of the model in terms of process outcomes (wait times for services, for example) [15,16]. Despite evidence demonstrating that we should always design and measure the impact of a MoC in terms of end-user experience [14], the extent to which rural MoC research has examined other aspects of impact for the end-user such as effectiveness, timeliness, efficiency, equity, patient-centredness, and safety has not been examined in depth. The concept of “quality care” as reported by the Institute of Medicine (IOM) encompasses these important impact measures as domains of quality [17]. High-quality healthcare, as defined by the Lancet Global Health Commission, involves consistently delivering care that improves or maintains health outcomes, is valued and trusted by all people, and responds to changing population needs [18]. The assessment of quality in children’s healthcare and comprehensive evaluations of rural and remote health services remain sparse [4,18,19,20,21]. Poor-quality care not only leads to adverse health outcomes but also results in wasted resources, economic losses, and decreased trust in health systems [18]. As such, this paper aims to (1) map the range of MoCs described in the evidence base that address child development needs in rural areas, (2) categorise them for the comparison of component parts and outcomes, and (3) assess their impact on children’s outcomes in relation to quality healthcare domains.

## 2. Materials and Methods

### 2.1. Study Design

This scoping review was conducted and reported in alignment with the Joanna Briggs Institute (JBI) methodology for scoping reviews [22] and the Preferred Reporting Items for Systematic Review and Meta-Analysis extension for Scoping Reviews (PRISMA-ScR) protocol [22]. Scoping reviews allow for the inclusion of both grey and black literature, data synthesis, and data mapping with greater breadth and depth as well as the ability to identify knowledge gaps to inform further research practice or policy [22]. 

The protocol for this scoping review was registered on Open Science Framework Registries (https://osf.io/tx6p8/).

### 2.2. Identifying Relevant Citations

The search strategy was developed by the authors with guidance and approval from an academic librarian and included terms relating to “children”, “allied health”, “development”, and “rural”. Following the initial scoping search through MEDLINE (File S1, MEDLINE search strategy), key terms and medical subject headings (MeSHs) were identified. This search strategy was modified for additional databases including EMBASE, EMCARE, the Cochrane Database of Systematic Reviews, Scopus, CINAHL, PsychINFO, and INFORMIT. 

A thorough grey literature search was conducted including Google School and the Google search engine (up to the first ten pages) as well as following the “Grey Matters” protocol [23]. In addition to this, theses were searched via Proquest and Trove, relevant research institutions (e.g., Australian Institute of Health and Welfare, Centre for Global Child Health Research, and Murdoch Children’s Research Institute), and other relevant peak bodies and networks (e.g., National Rural Health Alliance and Services for Australian Rural and Remote Allied Health). Content experts were contacted to identify additional organisations or literature that may be relevant to this review. The reference lists of relevant reviews and all included literature were also searched.

Searching was conducted in May 2023 and results were imported into EndNoteTM to screen for duplicates initially. All results were transferred into CovidenceTM and re-screened for duplicates and for screening to commence. 

### 2.3. Study Selection 

#### Inclusion/Exclusion Criteria

This scoping review considered both quantitative and qualitative research. Secondary research, including systematic reviews, was not included.

### 2.4. Population

Publications and grey literature were included if they investigated an allied health model of care that was designed to address children’s developmental needs in rural and remote areas. Citations were included if they provided original data on (1) MoCs for children with developmental needs and (2) reported findings beyond process outcomes (i.e., efficiency of resources, waitlist times) and included end-user outcomes such as clinical outcomes and patient experiences. Process outcomes were extracted from papers with end-user outcomes. Papers solely focusing on process outcomes were excluded. 

#### Allied Health Professionals

Allied health disciplines were selected based on the initial scoping search [15,24], first-hand experience of the authorship team, and discussions with end-users. Disciplines included were Physiotherapy/Physical Therapy, Speech Pathology, Occupational Therapy, social work, dietetics, psychology/behavioural specialists, Clinical Exercise Physiology, and Podiatry. We also included allied health assistants (AHAs) and university students supervised by these professionals. 

### 2.5. Context

#### 2.5.1. Rural and Remote Locations 

The Modified Monash Model (MMM) was used for clarifying rurality within Australian-based citations [25]. MMM 2–5 were included in this paper. Rural areas outside of Australia were based on the labelling of rurality within the citation. 

#### 2.5.2. Countries

For this review, the literature from only developed countries was sought. Given that MoCs from other jurisdictions (such as low- and middle-income countries) are likely to be different (to cater to local context and resourcing), the literature from these countries was excluded. Such approaches have been reported in previous literature [26]. Developed countries were defined as per the United Nations [27].

### 2.6. Concept

#### Models of Care

A MoC is a concept that is not consistently defined across the literature. For the purpose of this paper, we have used a definition prepared by Services for Australian Rural and Remote Allied Health [13] (SARRAH), which states that a model of allied healthcare is

*The distinct arrangement of service delivery that an allied health profession or professions adhere to and which is recognisable through an articulated philosophy underpinning the service, a set of overarching principles, and defined core elements that distinguish one particular method of service delivery from others at work in the system. A model of care is more than the individual modalities and technologies it uses to implement its service delivery*

*[13].*

Models discussed by SARRAH under this definition include services such as outreach models, sessional employment, centralised services, and disease-specific units [4,13,28]. Citations were excluded where they described individual interventions or assessment tools, rather than the complete arrangement of service delivery (MoC). Citations were excluded if they only investigated a single intervention without details of a MoC it was within, or if they did not report child health outcomes. See Table 1 below for detailed inclusion and exclusion criteria. 

### 2.7. Screening and Data Extraction

Title and abstract and full-text screening was conducted by two independent reviewers (G.G. and S.K./H.B.). All discrepancies between reviewers were resolved via discussion. Data from all included papers were extracted by one reviewer (G.G) and summarised in a piloted Excel spreadsheet. A second reviewer (H.B.) independently extracted data from 25% of papers (*n* = 7). 

### 2.8. Data Synthesis

Given the nature of this review, a narrative approach was taken to synthesise data. The Methods section of each citation, reporting on how a service was delivered, was used to determine components of MoCs. Following this, one research team member (G.G.) defined the component parts of and categorised MoCs into comparable models. Some citations were grouped into multiple categories as they used multiple strategies to deliver their services. 

The second stage involved the application of the IOM quality framework (File S2—definitions of IOM quality domains and subdomains) to each MoC paper [17]. A framework analysis [30] was applied, enabling the systematic extraction of information from citations using IOM quality descriptors as the framework [17]. The language and information contained in the Objectives and Outcomes sections of each paper were used to identify themes that would enable (a) the underpinning need for/purpose of each MoC and (b) the impact of the MoC on end-users/patients to be categorised into one or more IOM quality descriptors. This process also allowed for the identification and development of subdomains of IOM relating to impact. These have been reported in the findings (File S2). For example, the quality domain ‘effective’ was separated into two subdomains—Effective 1: improving screening, assessment, and diagnostic processes to ensure that services are delivered to those who are likely to benefit and not providing services to those not likely to benefit and Effective 2: evaluating the benefit of a service. (See File S2 for all subdomains.) These subdomains were developed as they increased clarity of how impact was reported in the outcomes of included citations. One reviewer (G.G.) undertook initial thematic coding and mapping with assistance from another research team member (A.M.). The themes were then verified by two other researchers (H.B. and S.K.). 

The findings of this stage were then combined with the findings from stage 1, categorisation, to enable comparisons of impact to be made across groups of MoCs. This stage involved all researchers with any discrepancies resolved through discussion and consensus.

## 3. Results

### 3.1. Study Selection

**Figure 1:**
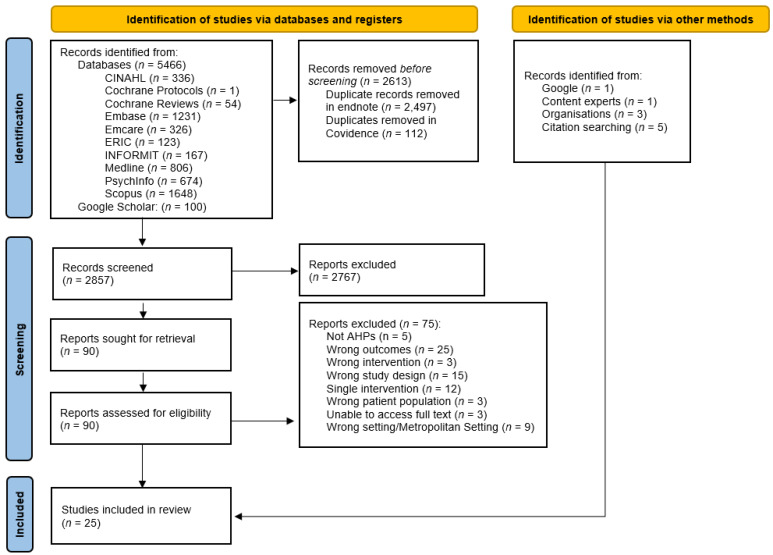
PRISMA flowchart.

The search identified 5466 citations from the literature. Following removal of duplicates, 2857 abstracts were screened. In addition to this, 106 grey literature sources were searched along with reference lists of the included academic literature, identifying a further 10 citations that satisfied inclusion criteria (Figure 1—PRISMA chart). From these, six were identified through citation searching [31,32,33,34,35,36], three from organisation websites [37,38,39], one from a content expert [40], and one from Google [41]. A total of 25 citations met the inclusion criteria.

#### 3.1.1. Population

A full review of the population involved in the included citations is available in Table 2. From citations that reported the number and age of children involved, a total of 13,283 children from birth to 18 years of age were observed. One citation did not report the number of children [39], and three citations simply reported participants as ‘children’ rather than a specified age [32,39,42].

Children were accessing or seeking allied health services for a variety of reasons (Table 2). Most frequently, these related to speech and language development concerns (*n* = 15) [31,32,33,34,37,42,43,44,45,46,47,48,49,50,51] or general developmental concerns (*n* = 6) [36,37,41,45,50,52]. Some reasons for seeking services were due to recommendations from other people from the team around the child (TAC) (school staff, families, other therapists, etc.), while others had established health conditions requiring intervention and therapy, such as foetal alcohol syndrome [50], autism spectrum disorder (ASD) [35,39,40,45,46], and cerebral palsy [50]. 

A total of nine different AHPs were involved. They included speech and language pathologists (SLPs) (*n* = 18) [34,38,40,43,44,45,47,48,50,51,52,53,54], occupational therapists (OTs) (*n* = 9) [34,36,37,38,40,41,45,50,52], physiotherapists (*n* = 5) [36,41,50,52,55], social workers (*n* = 4) [35,41,52,55], audiologists (*n* = 3) [37,39,42], psychologists (*n* = 3) [40,46,52], dieticians (*n* = 2) [37,41], podiatrists (*n* = 1) [41]. Three citations included AHP students delivering all therapy [31,32,33] and one citation involved AHAs [39]. Fourteen citations involved the children’s parents/carers in the delivery of therapy [35,40,42,43,44,45,46,47,48,49,50,53,54,55] and thirteen citations reported the involvement of education staff (e.g., teachers, teaching aides) in identifying children at risk or the delivery of therapy [31,33,34,40,41,42,43,44,45,47,51,52,53] (Table 2). 

#### 3.1.2. Context

Citations were included from five different countries: Australia, the USA, Germany, Taiwan, and Canada (Table 2). The classification of rural and remote varied between citations due to the range of countries included. Within the Australian context, no citations provided MMM ratings; however, for 11 citations [31,32,33,34,36,38,40,41,43,44,45,49], locations provided were interpreted using the MMM scale [25], resulting in the range of MMM level 2 to 7, meaning that all levels of remoteness were represented in results. Citations were conducted across a variety of contexts, most commonly, in education settings (*n* = 11) [31,32,33,40,41,42,43,44,45,49,50,51,53], teletherapy/online-assisted services (*n* = 11) [34,39,40,42,45,47,48,50,51,53,55], home services (*n* = 10) [35,36,39,40,45,48,50,52,54,55], clinic settings (*n* = 6) [35,36,46,49,52], and visiting clinics (*n* = 2) [37,38]. 

**Table 2:** Population and Context.

Study	Country	Study Design	Context	AHPs	Child Data	Others Involved	Parameters	Brief Elements
								
Australian Institutue of Health and Welfare [38]	Australia	Descriptive (report)	Visiting clinic	SLP OT Audiologist	*n* = 5938 Age: <18 years Dx: Screening for hearing concerns	Ear, Nose, and Throat doctors Nurses	Duration: 624 weeks (12 years) Frequency: One-off visit, 2× per year per location Comparator: Pre–post assessment of self	**Queensland Deadly Ears Program** Visiting clinic for screening and intervention services (intervention is surgical intervention). The program is for ear and hearing services for Aboriginal and Torres Strait Islander children from communities across rural and remote Queensland. Speech and OT services would consult as required. teleFIT was a service provided for hearing aides. AHPs within this service would refer to other services if children screened required ongoing services.
Autism Spectrum Australia 2021 [39]	Australia	Qualitative	Teletherapy Home visits Education settings	AHAs	*n* = not reported Age: not reported Dx: ASD	Therapists supporting AHAs	Duration: Not reported Frequency: Not reported Comparator: Nil	**AHA-Assisted Therapy** AHAs were provided with programs from the clients’ therapists. An interdisciplinary approach was therefore taken. The AHA provided the intervention services within the client setting. Goals were encouraging capacity building to allow the children to increase participation. Regular meetings between the AHA and therapist were provided to allow for support and adjustment of the program if necessary. Telehealth meetings occurred to monitor progress.
Bohlen, G. 1996 [52]	Germany	Descriptive (report)	Home visits Clinic	Physiotherapist OT SLP Psychology Social work	*n* = 224 Age: average, 40 months Dx: General developmental delay (34.4%) Language development disorder (25.9%) Fine and gross motor skill disturbance (17.9%) Impairments in perception and perception processing (11.6%) Play and contact behaviour problems (18.8%) Damage to sensory organs (11.2%) Brain damage (down syndrome, alcohol embryopathy) (5.8%) No diagnosis (5.8%)	Doctors Teachers	Duration: 6 months Frequency: 1× home visit for background 1 × 30 min assessment Comparator: Nil	**“The Early Detection Team”** Screening services to identify potential disability. Concerns were raised by parents/carers, doctors, or other members of the team around the child. The early detection team then provided a diagnosis if required and drew up a treatment plan or referred to a specialist if required. No formal developmental diagnostic tool was used; assessment was conducted in the structured game situation. Other members of the early detection team observed what was happening through a one-way pane and a video recording was made. Observations were discussed between the team and a home visit will communicate this to the family.
Chase et al., 2008 [42]	USA	Descriptive (report)	Education centres	SLP Audiologist	*n* = 51 Age: not reported Dx: Speech delays Hearing concerns/referred	Research team Parents/carers Teachers	Duration: 15 months Frequency: One-off assessment 5× parents’ education sessions Comparator: Nil	**Consultative Model** Speech, language, and hearing diagnostic treatment services delivered by speech-pathology and audiology graduates in an Appalachian Early Learning Centre. Parents or teachers accompanied children as needed during testing. Parents/carers were taught to use natural learning opportunities using adaptations of the Learning Language Together model.
Davies, S. 2007 [36]	Australia	Descriptive (book)	Clinic Home visits	Physiotherapist OT SLP	*n* ≥ 200 Age: <2 years Developmental delays in two or more areas—Cognitive/Gross motor/Fine motor/Communication development	Special educator Family support worker	Duration: 4 years Frequency: 30–45 min appointments Frequency varied depending on the child Comparator: Nil	**Rural Beginnings Project** Family-centred practice that utilised a transdisciplinary team approach. Children accessed this service if they had a developmental delay in two or more areas. Senior therapists are employed to provide support to other team members. This included diagnostics, treatment, and further referral to additional services if required.
Dettwiller and Brown 2015 [32]	Australia	Descriptive (report)	Education centres	Students	*n* = 46 Age: not reported Dx: Language and communication delays	Clinical supervisors	Duration: 2 years—3 cycles complete at the time of program completion Frequency: 8 weeks Comparator: Nil	**Speak Easy for Learning and Living** Service–learning delivery model that included cross-sectoral partnerships between universities, health services, school education, and the community. Groups of university students work under the guidance of a clinician academic to deliver services that include screening, assessment, treatment, and referral. Students are required to complete a comprehensive induction and orientation program. A six-week program/schedule was provided that students were to complete.
Dodd et al., 2019 [43]	Australia	Descriptive (report)	Education centres	Physiotherapist SLP Students	*n* = 114 Age: 4–7 years Dx: Late talkers Speech and language concerns Literacy delays	Teachers Parents/carers	Duration: 4 weeks intensive student-led clinics Frequency: Average of 6 × 30 min sessions Comparator: Nil	**Student-Led Model** Children were referred by teachers, parents, and local SLPs. This model involved SLP students to provide 4-week-long intensive clinics in schools. The students were provided with clinical supervisors from the local health department. Students used selected standardised paediatric assessments from the university and assessments that are commonly available in clinics.
Fairweather et al., 2016 [53]	Australia	Mixed methods	Education centres Teletherapy	SLP	*n* = 19 Age: 3–12 years (average, 7.8 years) Dx: Communication difficulties	Therapy assistant Volunteer parent Volunteer employee Teaching aides	Duration: 12 weeks Frequency: 6× fortnightly sessions Comparator: Nil	**“Come N See” Speech–Language Pathology** School-based teletherapy program. This used low-bandwidth technology and assessed the suitability of this technology. Face-to-face outreach assessments were initially conducted by Royal Far West. Children were referred/nominated by their school/preschool. Children who required further services were given a block of teletherapy services, following treatment goals that had been determined between the parents/carers, school staff, and treating SLP. The adults supporting the children were provided with remote, technology-based, therapy support to continue to provide the child with therapy-related activities.
Heins, K. 1998 [44]	Australia	Descriptive (report)	Education centres	SLP	*n* = 20 Age:4–7 years Dx: Screened for speech and language delays	Teachers Parents/carers Teaching aides	Duration: Approximately 40 weeks (one school year) Frequency: 3× sessions (assessment and two reviews) Ongoing support from teachers, parents, and aides as required Comparator: Nil	**Collaborative Consultation: In-School SLP Screening and Intervention Program** Screening services for speech and language concerns for children in schools. The process included identifying the problem and deciding on appropriate intervention over two sessions. Ongoing support was provided via a program delivered by teaching staff and parents. Teaching staff and parents were provided with skill development workshops to be able to assist in providing the intervention programs to children.
Hines et al., 2019 [45]	Australia	Mixed methods	Teletherapy Education centres Home visits	SLP OT	*n* = 4 Age: average, 6.38 years Dx: Speech and language delay Social, emotional, and motor planning issues Incontinence Comprehension Attention School preparedness ASD	Teachers Parents/carers Teaching aides	Duration: 12 weeks Frequency: 7–15 sessions Comparator: Nil	**Complex Disability Supports via Teletherapy** Teletherapy was used to deliver services for children with complex disability. Specific features of the service delivery model varied including the location of telepractice, participants attending telepractice sessions, and the number, duration, and frequency of sessions. Real-time, web-based video conferencing connected AHPs from their practices’ locations to a web-cam-equipped laptop or tablet in the child’s preferred location. Children were funded by the National Disability Insurance Scheme. Adults were required to attend sessions and were involved in designing how the service would be delivered for them.
Hoffman et al., 2019 [46]	USA	Descriptive (case study)	Teletherapy Clinic	Behaviour specialist	*n* = 4 Age: average, 2.063 years Dx: Challenging behaviour ASD Speech delay	Parents/carers Supervising BCBAs		**Teletherapy Parent Training** Behaviour specialists were trained over teletherapy by a board-certified behaviour analyst. These behaviour specialists then provided teletherapy training to parents so that they could provide a functional assessment of their child and engage in implementing procedures for intervention.
Hsieh et al., 2020 [55]	Taiwan	Pilot RCT	Home visits Teletherapy	Physiotherapist Social work	*n* = 24 Age: 6–33 months Dx: Gross motor delays	Parents/carers Paraprofessionals	Duration: 12 weeks Frequency: 4× biweekly visits in the first 2 months and single visits in the 3rd month Session length: 1–1.5 h per visit Comparator: Nil	**Collaborative Home-Visit Program** A pilot randomised control trial. The experimental and control groups received home visits. The concepts of transdisciplinary and interdisciplinary approaches. The physical therapists organised the intervention project and developed individualised service plans with local team members (social workers and direct service providers (DSPs)). The DSPs conducted home visits to instruct parents. Online case meetings that occurred fortnightly occurred to ensure the quality of home visits.
Jessiman, S. 2003 [47]	Canada	Descriptive (case study)	Teletherapy	SLP	*n* = 2 Age: 7 years and 5 years, 4 months Dx: Speech and language delays	Parents/carers School staff	Duration: 8 weeks Frequency: 2× weekly Comparator: Nil	**SLP using Regional Satellite-Based Telehealth System** Telehealth was used for SLP assessment and treatment. Assessed over camera and then three days later in person. To help with the audio difficulties, lapel microphones were purchased and sent to the remote site where the speech and language treatment would take place. Treatment options involved the development of an individual SLP program to be implemented by the parents or school personnel. After these were developed, the SLPs travelled to and from the community to explain the programs to parents and teachers. Follow-up was to occur at the school’s request but there was a lack of this due to a lack of personnel willing to carry out the programs.
Johnsson et al., 2018 [40]	Australia	Mixed methods	Teletherapy Home visits Education centres	OT SLP Psychology	*n* = 16 Age: 0–12 years Dx: ASD	Key worker School staff Local therapists Parents/carers	Duration: 52 weeks Frequency: 1 h sessions, 6× fortnightly Comparator: Nil	**Building Connections** Online interactive webinars were provided to AHPs. Capacity building. Teletherapist scheduled a session to inform a family support plan. Children were to be supported by a carer/teacher and local AHP could be involved. Children were provided with 6× fortnightly teletherapy sessions.
Jones et al., 2015 [31]	Australia	Descriptive (report)	Education centres	Students	*n* = 253 Age: 4–5 years Dx: Speech and language concerns	University staff School staff Teachers	Duration: Approx. 30 weeks (3 school terms) (data were from one year but the program has been running for at least six years) Frequency: Up to 20 sessions annually 6–8-week blocks of sessions Comparator: Nil	**Allied Health in Outback School Program (AHOBSP)** Children are referred by their teachers for this service if their teachers have concerns with language. University students provide screening, assessment, and therapy for children identified with mild to moderate needs. Children with complex needs were referred to hospital-based clinicians. University students rotate every 6–8 weeks.
Kirby et al., 2018 [33]	Australia	Pre–post	Education centres	Students	*n* = 122 Age: 4–6 years Dx: Speech and language delays	Teachers	Duration: 12 months Frequency: 3.3, 6.2, and 7.9 sessions on average for children with mild, moderate, and severe delay, respectively Comparator: Nil	**Service–Learning Program (Student-Led Clinic)** For each child, students provided screening and assessments and made plans for treatment if indicated. Assessment after screening confirmed the screening findings and indicated the appropriate therapy. Screening sessions were arranged to fit with school curriculum requirements and children’s attendance at school, to minimise waiting times. Children were referred to local services at the end of the program.
Langbecker et al., 2019 [34]	Australia	Pre–post	Teletherapy	OT SLP	*n* = 98 Age: reported as prep to grade 6 Dx: Speech and language delays, Educational and participation in class concerns	Teachers Teaching aides	Duration: 12 weeks Frequency: 1× weekly Comparator: Nil	**Health-E Regions: Telehealth Service Model** The telehealth service offered SLP and OT via video conferencing to children at five rural Queensland schools teaching at least grades prep (the first year of schooling in Queensland) to grade six. At the beginning of each semester, children were chosen for participation in SLP and/or OT via telehealth following local processes at each school, including identification of problems, assessment for suitability, and consent by parent/guardian. Selection was not by formal clinical diagnosis; however, some children may have had a prior diagnosis of a speech/language disorder.
Lim et al., 2020 [48]	Canada	Mixed methods	Teletherapy Home visits	SLP	*n* = 4 Age: 4 years, 2 months–7 years, 2 months Dx: History of speech delay—met criteria for childhood apraxia of speech (CAS).	Parents/carers	Duration: 10 weeks (2× 4-week blocks (2-week break in between)) Frequency: 15 min, 2× per day, 5× per week Comparator: Nil	**Parent-Led Dynamic Temporal and Tactile Cueing (DTTC)** Four parent training sessions, which included online and offline sessions. A manual was provided to assist with the treatment protocol. This parent training program used DTTC to improve the speech skills of children with CAS living in a remote location. During the treatment phases, parents were asked to provide treatment at home for 15 min, twice a day, five days a week. A DTTC board game was designed and provided to each parent to help when working with their child.
Mathisen et al., 2016 [49]	Australia	Qualitative (phenomenological)	Education centres	SLP	*n* = 10 Age: <3 years and between 3 and 5 years Speech and language concerns	Parents/carers	Duration: 6 months Frequency: One-off Comparator: Prior knowledge	**Talking Matters Bendigo (TMB)** A walk-in education clinic aimed to develop parents’ skills and education in supporting their child’s language development. This clinic allowed for observations of a child to be made by an SLP. This service did not provide diagnoses. Where necessary, the SLP would provide some simple recommendations such as linking the child back to universal services for further global development assessment as required, referral to another health professional, provision of simple ideas to encourage speech or language development at home, and/or provision of a simple hand-out, similar to the Hanen programme, ‘It Takes Two to Talk’.
Nevada Department of Human Services 1997 [50]	USA	Descriptive (report)	Education centres Home visits	Physiotherapist OT SLP	*n* = 1193 Age: 0–6 years Dx: Erbs palsy FTT Language delays Bilateral haemorrhage, developmental delays Early birth, developmental delays General developmental delays—absence of formal Dx Poor social environment Speech delays Prenatal exposure to gonorrhoea Facial anomalies FASD NICU stay Ectopic anus Perinatal drug exposure Foster care—abuse and neglect at home Apnoea Trisomy 22 Cleft palate CP Down syndrome Viral meningitis at 3 weeks	Parents/carers	Duration: Not reported Frequency: Computer-assisted curriculum to be completed as prescribed Monthly home visits Yearly assessments Comparator: Nil	**“HAPPY Rural Outreach Program”** A specialist was able to provide monthly home visits. This required the parent’s participation in the assessment of the program development. Children were assessed using the Developmental Programming for Infants and Young Children Scale. A computer-based curriculum was delivered alongside service coordination of additional therapies, along with consultative therapies. Recommendations and consultations were all videotaped.
Royal Far West 2022 [37]	Australia	Qualitative	Visiting clinic	OT SLP	*n* = 4371 Age: 3–5 years Dx: Children screened for child health, oral, hearing, dietetics, speech and language, and fine/gross motor development	Nurses	Duration: 73 weeks (six years) Frequency: One-off Comparator: Nil	**“Healthy Kids Bus Stop”** The HKBS delivers a comprehensive health screening in line with the NSW Health “Child Personal Health Record” (Blue Book). The health screening is undertaken by a multidisciplinary team of nursing and allied health staff from Royal Far West, working with staff from other agencies such as the Local Health District (LHD), the Primary Health Network (PHN), Aboriginal Health Services, and other local health service providers. At the conclusion of the day, a multidisciplinary case conference is undertaken where each child’s health screening is reviewed and used to develop a coordinated referral pathway. The pathway includes the child’s local GP and Child and Family Health Nurse as key referral points, with Royal Far West, the Local Health District, Aboriginal Health Service, the Primary Health Network, and other local services supporting the child’s identified health needs.
Short et al., 2016 [51]	USA	Pre–post	Education centres	SLP	*n* = 578 Age: 3–18 years Dx: Speech and language concerns	Paraprofessional (education)	Duration: 80 weeks (2 school years) Frequency: 2× in-person assessments 1–2 times per week using real-time two-way interactive teletherapy Students were seen 36.6 ± 0.6 min per week in 2012–2013 (range: 3–60 min) and 41.3 ± 0.8 min per week in 2013–2014 (range: 5–60 min) Comparator: Compared to NOMS (onsite) database	**Speech Teletherapy** Speech teletherapists completed onsite school visits at the beginning and end of the school year to conduct evaluations, review records, and meet parents and school staff. All other treatment sessions, typically once or twice per week, were conducted using real-time and two-way interactive video telecommunication technology between the INTEGRIS Health metropolitan site and the respective rural schools.
Swift et al., 2009 [54]	Australia	RCT	Home visits Teletherapy	SLP	*n* = 29 Age: 2–12 years (average, 7 years) Dx: Conduct problems—TOOL: Therapy attitude inventory and Eyberg Child Behaviour Inventory	Parents/carers	Duration: 12 weeks Frequency: Self-guided Weekly telephone support approx. 2 h a week Comparator: Waitlist control	**Telephone-Guided Parent Training Program** A randomised controlled trial investigating AHP delivered a telephone-guided version of a parent training program (*Defiant Children*). A self-help book and workbook were provided with parents receiving evaluation questionnaires pre- and post-intervention. Parents were provided with regular access to support, which included a free call number to access the AHPs on a weekly basis and if they did not call themselves, they were followed up with fortnightly.
Turner-Brown et al., 2016 [35]	USA	RCT	Home visits Clinic	Social work	*n* = 50 Age: 29.6 months (intervention) and 29.7 months (control) Dx: ASD	Parents/carers	Duration: 6 months Frequency: 20, 90 min in-home sessions Comparator: Services as usual (SAUs)	**“Family Implemented TEACCH for Toddlers” (FITT)** In-home sessions (20) with an additional four clinic-based family sessions. In-home and parent sessions were combined to provide parent support, psychoeducation, and parent coaching. FITT is a parent education and support intervention designed to assist families in (1) better understanding how autism may be impacting their toddler, (2) how to better engage their toddler throughout the day, and (3) how to implement Structured TEACCH steps.
Williams and Healy 2007 [41]	Australia	Descriptive (report)	Education centres	Physiotherapist OT SLP Podiatrist Dietician Social work	*n* = 136 Age: 0.5 years–5 years Dx: Screened using development screening test for development delays	Program director Preschool coordinators	Duration: Not reported Frequency: 15 min, 2× yearly Comparator: Nil	**Busy Bee Screening** Multidisciplinary screening of children under 5 years. Screenings were held in kindergartens in local towns. Both locally based and visiting professionals were involved in the service. The Australian Developmental Screening Test was used to screen children. A parent is given the form and rotates through appointments with varied professionals. On completion of each section, the parents were given a verbal indication of results and recommendations. A report is created with recommendations from each professional and sent to the families.

USA—United States of America; AHP—Allied Health Professional; Dx—Diagnosis; OT—Occupational Therapist; SLP—Speech and Language Pathologist/Therapist; FTT—Failure To Thrive; FASD—Foetal Alcohol Spectrum Disorder; NICU—Neonatal Intensive Care Unit; CP—Cerebral Palsy; ASD—Autism Spectrum Disorder; BCBA—Board-Certified Behaviour Analyst; AHA—Allied Health Assistant.

#### 3.1.3. Concept

##### **Mapping Models of Care** 

The framework analysis allowed for the mapping of component parts of MoCs and resulted in the identification of four categories of MoCs: screening services, consultative services, role substitution, and online services (Table 3). 

##### **Screening Services** 

Screening services, where assessments were conducted to identify those with needs, and direct appropriate referrals or deliver treatment, were used frequently (*n* = 9) (Table 3) [31,32,33,37,38,41,50,52,53]. Screening services were conducted by local [31,33,41,49,50,52,53], visiting [37,38], or a combination of local and visiting [32,33] professionals, conducted within a variety of contexts. Children were referred to these services either by self-referral from parents/carers [37,38,41,52] or following a recommendation from education staff [31,33] or local health services [37,38] and within some studies, this was unclear [32,50,53]. Referrals that were sent on from screening services were most commonly to SLPs [37,41]; two others reported referrals to tertiary hospitals for more intensive treatment [31,38] and others to local community services [32,33,52]. 

##### **Consultative Services** 

Consultative services acted to provide education to local communities including AHPs, parents/carers, and education staff. This was performed in three different ways. Firstly, one citation engaged an education clinic that was provided to increase health promotion within a rural town [49]. This allowed for the education of community members, mainly parents, to increase their skills in supporting their child’s speech and language development. They could then be provided with simple recommendations, such as linking them to services for further assessment. Secondly, AHPs were upskilled by experienced AHPs to deliver specialised services. This included using teletherapy to educate behaviour specialists [46] or an autism specialist [40]. Finally, the team around the child (TAC) was provided with education to enhance their knowledge or their skills when interacting in daily life with the children. An example of this was a model that provided rural families with education on ASD to allow the parents/carers to understand the diagnosis better and how to manage it at home [35]. 

##### **Role Substitution** 

Differing from consultative models, role substitution requires the use of someone to take on the role of what would be expected of another discipline. This included transdisciplinary models, where AHPs would take on roles outside of their expected roles (e.g., SLP supporting parents to address waitlist clients for psychology and behavioural needs [54]) or student AHPs taking on clinics [31,32,33,43]. The use of AHAs allowed for therapists to deliver review-based services, while the AHA was able to deliver regular therapy and support [31]. This could also occur via the TAC taking on the role of the therapist. The TAC would often be provided with support and a program from the therapists and would then be expected to deliver ongoing therapy in replacement of the therapist. Some examples include school support officers delivering SLP programs [34,44,45,51,53] or parents/carers assisting in the delivery of a functional assessment [46].

##### **Online-Based Services** 

Online-based services, delivered as a typical clinic-style service via the internet or teletherapy methods, were used in three citations [34,45,51]. Typical services were considered as services delivered within the scope of the AHP providing the service, delivered directly to the child. Teletherapy was used by nine services to support a different MoC [39,40,46,47,48,50,53,54,55]. 

### 3.2. Quality Outcomes 

Collectively, findings were mapped across five of the six IOM quality domains: timely, effective, equitable, efficient, and patient-centred (safety was not measured or explored by any citation). The framework analysis allowed for both the development of subdomains for each of these and the categorising of outcomes under relevant subdomains of quality. Within the domains, we identified 12 subdomains related to quality of healthcare (File S2) (Table 4). 

#### 3.2.1. Effective

Effectiveness was the most reported domain (*n* = 22, Table 4). Findings associated with effectiveness were identified across two subdomains (File S2), with outcomes from one citation crossing both subdomains [38].

**Effective 1:**  *Improving screening, assessment, and diagnostic processes to ensure services are delivered to those who are likely to benefit and not providing services to those not likely to benefit (n = 5).*

All five citations [31,37,38,41,52] related to Effectiveness 1 used screening services as a MoC, with two of these citations also using role substitution [31,37]. Across these five citations, children were screened for general developmental [37,41] and hearing concerns [38] as well as specific speech and language concerns [31]. One citation using a screening service reported that 80% of children screened (*n* = 4371) were referred for ongoing developmental support from a variety of AHPs [37]. Another citation [31] that utilised students as a form of role substitution reported that 71% (*n* = 181) of children screened by SLP students required intervention and further student SLPs’ support. Most citations reported their outcomes as positive; however, as results were not compared to ‘no care’ or ‘standard practice’ (i.e., how many children would have been screened without the service), it is unclear how impactful these MoCs were. Qualitative data demonstrated that multidisciplinary approaches to screening enhanced the depth and breadth of assessment [45,47]. One citation reports that parents/carers viewed that the different perspectives from the multidisciplinary team ‘lead to a more holistic view of the child [52]. Overall, the use of screening MoCs with multidisciplinary teams and role substitution methods seems to improve screening, assessment, and diagnostic processes, leading to the appropriate identification of children who require services (Table 4).

**Effective 2:**  *Evaluating the benefit of a service (n = 18).*

Eighteen citations evaluated the benefits of allied health services [1,4,6,16,17,20,23,24,25,26,27,28,30,32,33,46,49,52] (Table 4). Of these, five citations used screening services [32,33,38,50,53], seven used consultative services [35,39,40,46,48,54,55], ten used role substitution [32,33,35,40,43,44,46,47,54,55], and ten used online services [34,39,40,45,46,47,48,51,54,55]. Child health outcomes were reported for speech, language, and communication (*n* = 8); social skills (*n* = 5); goal attainment (*n* = 2); handwriting/academic skills (*n* = 3); motor skills (*n* = 1); general participation in daily activities (*n* = 1); hearing/ear health (*n* = 1); and need for further therapy (*n* = 2). Of the citations evaluating the benefits of service, most reported positive impacts on child health [32,33,34,39,43,44,45,46,47,48,51,53,54], with four finding neutral or unclear findings [35,40,50,55]. Only four studies reported statistically significant results [34,35,54,55]. Positive impacts of screening services ultimately included an 18% reduction in ear conditions [38], improved speech and language progress in 82% of participants [53], qualitative evidence of children “using more words” [32], and further referrals to local services [33]. Consultative services provided positive benefits to child development in a variety of areas, including a statistically significant increase in daily activities [55]; a non-statistically significant trend for improved gross motor skills [55]; 33% of children achieving a “much greater than expected change” on the Goal Attainment Scale [40]; moderate effects on language skills [48]; a reduction in problem behaviours in a small group of children (*n* = 4) [46]; a statistically significant improvement in ASD symptom severity [35]. Qualitatively, it was reported that this MoC improved participation as an AHA reported that a child was now able to “[attend] childcare, she’s got friendships…” [39]. A combination of consultative services and role substitution reported a positive impact on communication with children with ASD [35] and an RCT [54] also utilised both these MoCs and demonstrated a statistically significant improvement in child behaviours for the intervention group. Another citation using role substitution reported that 17% of children required ongoing therapy once their service was finished [43]. Online services were reported to provide benefits within areas including handwriting and academic skills [34,35,45]; speech and language skills with improved language outcomes [51]; speech and language goals qualitatively reporting on progress [47]; and participation qualitatively reported to improve skills such as toileting and social engagement [39]. As depicted in Table 4, overall, for the citations related to the effectiveness of services, a positive impact on child developmental health was demonstrated for allied health services delivery via different MoCs, across a variety of contexts. Due to the large variety in how these were measured, this was difficult to quantify. 

#### 3.2.2. Equity

A total of 19 individual citations reported on equity in their outcomes (Table 4). Three subdomains of equity were identified (File S2).

**Equity 1:**  *Improving accessibility of services (i.e., reducing travel times, improving presence of services in areas) (n = 13) (Table 4).*

Accessibility of services was addressed in thirteen citations [31,34,35,36,37,39,40,43,44,45,46,47,53]. MoCs used in these citations included screening [31,37,53], consultative services [40], role substitution [31,36,37,39,40,43,44], and online services [34,39,40,45,47,53]. Eleven citations reported positive outcomes relating to improving accessibility [31,34,35,36,37,39,40,43,44,46,47] with two reporting neutral results [45,53]. Screening services, explored in three citations, were reported to be able to “fill gaps” by providing a service that was not there in the first place [31,37,53]. An RCT trialling a home-visit, parent education program reported that their program was “socially acceptable” in providing a service where families may not be able to access trained providers [35]. Online services were qualitatively reported to improve “practicality and convenience” due to decreased costs of travel, time, and losing schooling [53]. Across citations trialling online services, removing travel requirements was reported on favourably for increasing families’ abilities to access services as well as online services being able to increase flexibility and community awareness [36,39,40,45,53]. One citation utilised online services to access more intensive treatment for children with more significant problem behaviours and reported that the child continued to benefit from more typical early intervention services following this [46]. Involving members of the TAC through online services allowed for improved communication, increasing consistency of support for children [45]. Contrastingly, one citation noted that an area for improvement in this service, providing online SLP services, was to improve the communication between various stakeholders [53]. Another role substitution method, using an AHA service, reported that their services allowed families to “get the help [they] need” with parents reporting that “We had no one” [prior to the service] [39]. This citation, however, reported teletherapy to only be useful for “meetings, for collaboration, discussions with parents…” [39]. The use of alternate modes of delivering services, including transdisciplinary consultative or role substitution approaches, is beneficial in improving the equity of services. However, when these services are employed, communication appears to be of importance to parents/carers.

**Equity 2:**  *Increasing capacity of AHPs in rural areas (to ensure that the workload of rural practitioners does not vary from those in urban areas) (n = 4) (Table 4).*

All four citations reported positive outcomes relating to the capacity of AHPs [39,40,41,51]. MoCs used in the citations included screening [41], consultative services [40], role substitution [39,40], and online services [39,40,51]. A screening service provided in rural South Australia [41] resulted in appropriate referral services, which allowed for some children to be removed from waiting lists for services as once they were screened, they no longer required the service. Another approach, using online services to enhance capacity building, allowed for increased collaboration within the community, resulting in teletherapists being able to support local therapists [40]. The use of role substitution meant that therapists were supported through the use of AHAs to take on regular client support in replacement of the therapist [39]. Not only did this reduce the load on the therapist, but it resulted in positive reports from families including more consistent services and less travel [39]. Improving the capacity of AHPs was not commonly or explicitly reported in outcomes; however, through the use of role substitution of AHAs and online services in particular, local therapists were able to be more supported through mentoring from senior therapists.

**Equity 3:**  *Ensuring a high standard, and reducing the variability of healthcare irrespective of the location (n = 5) (Table 4).*

Providing a standard of care irrespective of location was reported in outcomes of five citations [32,46,48,49,50]. Included citations reported on MoCs that encompassed screening services [32,49,50], consultative services [46,48], role substitution [46], and online services [46,48,50]. An element of improving the standard of care involved improving the confidence and skills of AHPs [48,49,50]. One citation reported that because of the intervention program, the therapists were better able to provide parents with information [50]. Other citations reported success in relationship building and development of skills as well as therapists feeling empowered [48,49]. An addition to confident therapists was to have confident and skilled students. One citation reported that their service delivery methods, using screening services, resulted in increased confidence of SLP students and independence as a clinician [32]. The use of training parents and online services also allowed for access to more intensive and specialised services that would not have been available due to the location [46]. In one citation, providing parents with intensive treatments via teletherapy reduced their child’s problem behaviours [46]. One service provided a walk-in clinic for speech and language services [49]. This service assisted with providing more awareness and accessibility of speech and language services [49]. The standard of care of many services prior to the new MoC was not reported throughout the literature. However, increasing access to specialist services and increasing the skills and confidence of services already available was considered as ensuring a standard of care. 

#### 3.2.3. Patient-Centred

A total of 17 citations reported on outcomes relating to patient-centredness (Table 4). Three subdomains of patient-centredness were identified through synthesis (File S2). Four citations reported on more than one subdomain of patient-centredness [41,45,49,55].

**Patient-Centred 1:**  *Improving health literacy so that patients are able to appropriately advocate for and receive healthcare that aligns with their needs and values (n = 6) (Table 4).*

Six citations reported outcomes relating to addressing health literacy and engaged all MoCs: screening services [38,41,49], consultation [42,55], role substitution [36,55], and online support for services [55]. Elements of health literacy were measured by parents’ confidence in understanding their child’s condition as well as engagement with the service. Only half of the citations reported positive results relating to improving health literacy [36,38,41], with the other three citations reporting neutral or insignificant findings [42,49,55]. A pilot RCT [55], trialling a program that was monitored through home visits and online support, reported the perceived helpfulness score of their program (higher scores indicating higher helpfulness): average = 4.1 (SD = 0.51; range = 3.0–5.0). However, this pilot RCT also reported that there were no significant differences reported in parental knowledge between the intervention and control groups [55]. Another citation used a parent education program following recommendation for children to receive speech and language services, which resulted in 15/16 parents taking part in the program; however, only 1/16 parents engaged completely with the program [42]. In one citation [49], all participants (*n* = 10) reported that they had “no idea” what to expect from an SLP prior to the service but following the service, they had improved confidence and awareness of how to best support their child. Finally, another citation [41] reported that there was increased community awareness of AHP services just from the presence of the service. Health literacy was reported to improve only through the use of screening services and role substitution.

**Patient-Centred 2:**  *Including family and child perspectives of the service to ensure patient values are guiding clinical decisions (n = 11) (Table 4).*

Eleven citations involved parents in the review process and utilised screening services (*n* = 6) [33,37,41,49,50,53], consultative services (*n* = 3) [40,54,55], role substitution (*n* = 5) [33,37,39,40,55], and online services (*n* = 7) [39,40,45,50,53,54,55]. Eight citations reported positive outcomes while three citations [40,45,53] reported neutral or insignificant findings. Multiple studies reported “parent satisfaction” or “parent confidence” as an evaluation post [49,54,55]. Six citations reported qualitative parental reports [39,41,45,49,50,53]. Reasons for parental satisfaction included the following: reduced travel and cost of services [53]; consultative styles of therapy were reported on favourably with comments such as “consultation from therapists helps me to know what to work on with my child” [50]; parents felt more confident and empowered and “[providing] an approachable professional” [49,53]; parents were able to identify concerns better [41,50]; and working within the child and family’s environment was useful [45,50]. Technology was qualitatively reported as a concern with some parents reporting a lack of confidence when using technology required for interventions [53] and others reporting that “being remote, you don’t always have the best internet. Sometimes the internet was slow…that can cause problems” [45]. Only one citation [39] included child perspectives. This was completed using photovoice qualitative data where children were able to draw their experience and progress with their AHA as well as share photos [39]. Children shared pictures that depicted tools they had utilised such as whiteboard lists and “zones of regulation” [39]. A variety of parent-reported data were included amongst citations with varied reports of confidence with the most reduced confidence reported due to technology use. However, children were scarcely included in reporting, with only one citation carrying out this [39].

**Patient-Centred 3:**  *Co-designing and co-delivering services with families so that they are responsive to the family’s needs (n = 7) (Table 4).*

A total of seven citations included families and children in the development and delivery of a service [35,40,45,46,48,53,55]. MoCs included screening services [53], consultative services [35,40,46,48], role substitution [35,40,46], and online services [40,45,46,48,53]. Two citations used the Goal Attainment Scale where individualised goals for the program are developed, requiring participants to identify their own areas of concern [40,53]. In one citation, 78.9% (*n* = 15) children achieved at least one goal and 42% (*n* = 8) children achieved all of their goals [53]. Home visits were commonly used to set up programs for parents to complete [35,40,45,48,55]. An RCT [35] engaged parents by having one parent receive coaching to address their goals and given tools to support their child with ASD better at home. A pilot RCT [55] conducted home visits to monitor an individualised family plan. Consultative services that used parental education to provide ongoing support at home were a common feature of citations addressing this subdomain [35,46,48,55]. This resulted in outcomes including improved quality of the home environment (via the DA-IT-HOME, *p* < 0.0001, with a large effect size of 0.64 at follow-up) [55] and parents qualitatively reporting improved relationships with their child, “the time we do get together we cherish it” [48], and improved confidence [40,48]. Improved relationships were also qualitatively reported between children and the therapist, reported by a citation that used parents to support the child during teletherapy sessions [45]. The citation reported comments including “she’s [teletherapist] a lot more attuned to my son and what his needs are” [45]. Whilst challenges were reported in this process, parents were included in developing goals to direct the service and were often used to assist in delivery throughout citations, resulting in improvements in quality relating to patient-centredness. 

#### 3.2.4. Timely

Twelve citations used timeliness to describe the impact of a MoC (Table 4). There were three subdomains identified as specific to timeliness (File S2). Two citations reported outcomes associated with two subdomains of timeliness [33,44].

**Timely 1:**  *Improving referral pathways to ensure people receive appropriate care (increasing access to receiving a referral and decreasing unnecessary referrals) (n = 7) (Table 4).*

Seven citations reported on improving referral pathways [31,33,37,38,41,42,52]. Most citations described the use of screening services (*n* = 6) as well as role substitution (*n* = 3) and finally consultative services (*n* = 1) as the primary MoC. Four citations [37,38,41,52] included the use of a multidisciplinary team, to allow for role substitution, and three citations engaged student clinics as a form of role substitution [31,33,42]. All citations reported a positive impact on referrals. This could have been through a reduction in referrals through children being seen or an increase in outgoing referrals (due to increased appropriateness of referrals). Hearing specialists were referred in two citations with one citation referring 11 children for additional hearing services [42] and another citation referring 1492 children to a range of hearing specialists over a four-year period [38]. Royal Far West’s [37] Healthy Kids Bus Stop screening services referred 3500 children for further allied health assessment. A citation utilising student-led clinics reported that 101 children from 122 screened were able to be identified as requiring further treatment from students and 12% of these were referred for further services [33]. There was no historical data to allow for comparisons of how many children would have been screened without these services. Subsequent referral data were not reported. In addition to increasing access to referrals, one citation reported qualitatively that the use of a screening clinic meant that “the diagnosis and support plan receive a better foundation” [52]. Qualitative support for improving referral pathways through multidisciplinary teams using role substitution involved the statement that “much is to be gained from multidisciplinary assessments… to identify the most appropriate referral options and pathways for children living in rural and remote areas” [37]. Another citation included parental reports with positive evaluations from the parents receiving the service [41]. Referral pathways were improved through the implementation of role substitution methods, multidisciplinary teams and student-based services, and screening clinics. However, where referral numbers increased, the impact of this was not reported, that is, whether children were seen or were required to wait lengthy periods.

**Timely 2:**  *Increasing availability of professionals to reduce delays of those who give care (n = 5) (Table 4).*

The availability of AHPs was discussed in the outcomes of five citations [33,36,43,44,51]. This subdomain was addressed using role substitution (*n* = 4) [33,36,43,44], screening (*n* = 1) [33], consultative services (*n* = 1) [36], and online services (*n* = 1) [51]. Role substitution MoCs utilised a transdisciplinary approach [36], AHP students [33,43] and school staff [44,51]. All citations reported positive impacts on this subdomain. One citation reported that despite there being limited resources in the area, role substitution enabled better help for school children with speech and language needs [44]. Quantitatively, it was reported in a citation that there was an improvement in efficiency of therapy time that was spent to achieve clinically meaningful advancements in treatment goals as per the Functional Communication Screening Tool [51]. Another important result reported qualitatively that the use of role substitution reduced travel times for families to see appropriately skilled AHPs [36]. This was achieved through in-person consultation with a senior therapist to support other team members [36]. Another citation that engaged the use of SLP student placements reported that the use of students increased the availability of therapy, resulting in 83% of children screened receiving a service and ongoing goals for the child [33]. There were reports of high satisfaction with these methods, with parental support of up to 100% [33]. Overall, role substitution was most commonly reported to improve the availability of health professionals and offered more efficient therapy time, reduced travelling, and an increased number of children provided with therapy.

**Timely 3:**  *Reducing waiting lists to prevent harmful delays (n = 2) (Table 4).*

Only two citations included outcomes relating to the subdomain of addressing waitlists [44,54]. These citations used role substitution [44,54], consultative [54], and online services [54]. An RCT trialled the use of a 12-week program that involved parents and SLPs to deliver services while supported by behavioural specialists to provide services for children with behavioural needs who had been on waiting lists for significant periods [54]. Reported outcomes were children being seen by an AHP within three months in comparison to the expected 3–12-month waitlist [54]. Increased liaisons between health professionals, school staff, and parents were also reported [54]. The second citation [44] substituted ongoing therapist support with ongoing support from education staff to reduce waitlist times. Education staff received training from SLPs via skill development workshops to deliver speech and language programs [44]. Reduced waiting times for children identified as high priority (e.g., more significant speech and language concerns) were qualitatively reported [44]. SLPs positively acknowledged the benefits of the program, and teachers reported that the skills they learnt would benefit other children in the classroom [44]. Overall, role substitution appeared to reduce waitlist times when delivering services outside of the clinic. 

#### 3.2.5. Efficient

Three citations reported on efficiency in their outcomes (Table 4). Only one subdomain was identified (File S2).

**Efficient 1:**  *Optimising the use of resources (human, financial, and infrastructure) to avoid waste (Table 4).*

Three citations [42,47,51] reported on efficiency and used a variety of MoCs including screening [50], role substitution [47], and online services [47,50,51]. One citation [50] provided materials that could be used as an ongoing resource that could train new staff due to high levels of turnover [50]. The availability of this same curriculum allowed for an increase in parent volunteers and engagement [50]. Two citations [47,51] provided support for shorter treatment sessions. One citation [47] quantified that it was appropriate for sessions to last 20–40 min for younger children compared to 30–60 min. This citation also discussed the requirement of appropriate equipment for speech and language therapy sessions to ensure that over teletherapy, delays in audio and visuals are minimised [47]. The requirement of equipment was noted as a challenge and potential negative impact on the efficiency of a service. Overall, citations reported positive effects on efficiency; however, efficiency was reported sparsely throughout the literature.

## 4. Discussion

This scoping review has systematically mapped allied health MoCs for children with developmental health needs in rural and remote areas in developed countries and assessed their impact on the quality of healthcare received by end-users/patients. This review identified a variety of MoCs, which were mapped against the IOM quality domains. Collectively, irrespective of the MoC, the evidence supports a range of positive outcomes for children with developmental concerns, delays, and disabilities in rural and remote areas. A particular issue of concern, however, was the limited evidence that included end-user experiences in these areas, despite this being a best practice recommendation for MoC design and development [14]. 

### 4.1. MoCs for Child Development Allied Health Services

There is increasing interest in child development allied health service delivery, due to the transition in healthcare away from acute illness to chronic long-term conditions [56]. This, however, is more challenging in rural and remote areas due to a lack of access to services [4,29,56,57]. As a result, numerous MoCs have been used throughout the literature and through the framework analysis, we were able to map findings into four main styles of MoCs. The most common MoC identified was role substitution, which, despite positive findings across all subdomains of quality, presents some practical challenges. First, there may be limited access to health professionals who are available and have capacity and capability to deliver education to other professionals. Second, school staff, who are already overburdened with their routine duties, need to have the time, resourcing, and support to deliver the service. Families also need to have the time and resources to deliver these services when they may be experiencing carer fatigue. In addition to this, there are challenges with children’s engagement [48] and accurate outcome reporting when parents deliver therapy, with mixed positive findings for this method of therapy delivery in the literature [58]. It is also important to consider the health literacy, emotional capacity, and motivation of non-AHPs trying to deliver services and whether it is appropriate to have others deliver therapy services. The use of role substitution, however, is common in rural areas including allied health services for medical services [59], general practitioners being trained by specialists to deliver more specialised services in chronic care services [60], and physiotherapists delivering musculoskeletal services in replacement of general practitioners [61]. 

Online services allow for more flexibility in the location of therapy, reducing travel time and improving the comfort of a family to receive the service in their own environment [16]. However, due to the isolation of rural areas, there were often reports of technology difficulties such as not having appropriate equipment or inadequate internet connection [45,53]. This is also consistent with existing literature, which identifies reported issues around technology and internet capabilities as considerations [62,63]. Consultative services, primarily aimed at upskilling those working closely with the child in their local community, were less commonly employed as a MoC. Positive results were yielded where motivation for the service came from those who received the support or education. One example within our included citations [31] noted positive results across all addressed domains of quality and had school principals approaching the local Department of Health to ask for assistance with managing children with SLP needs. However, in another citation, where the TAC was not voluntarily involved from the start, there were difficulties in obtaining required engagement [42]. This suggests that the usefulness of this type of service is reliant on the individual/family driving the service need. Similarly to role substitution, there are several reasons relating to the TAC that may make this service inappropriate, potentially resulting in decreased capacity to engage with these services. 

Finally, screening services allowed for more children to have developmental concerns identified. This service allowed for increased referrals, and improvement in quality of services, likely due to more appropriate referrals and increased health literacy and inclusion of families in the process. Screening services are used in the literature for adult services for conditions such as breast cancer [64] and diabetic retinopathy [65]. Included citations also report similar positive outcomes with a number of people appropriately identified as having these conditions. However, there was concern regarding a lack of reporting as to whether the increased number of children screened and referred were then able to access follow-up services and if so, whether these services were able to handle the increase in referrals. The scarcity of information regarding actions from referrals necessitates a careful consideration for a variety of reasons. First, from the perspective of children and their families, insufficient or delayed responses can heighten anxiety and concern about the commencement of necessary services [66]. Delays in timely actions could lead to poorer developmental outcomes [12]. Second, when referrals rise, there is additional pressure on already burdened rural AHPs. Lastly, more referrals require extra resources for a sector already facing significant resource constraints. Whether such consequences actually occurred is unknown, due to a lack of referral outcome data, but are a distinct possibility. 

### 4.2. Quality of Child Development Allied Health Services 

It has been discussed that a key tenant of successful MoCs is that they are designed and implemented with a focus on end-user needs and experiences, rather than organisational needs [14]. Therefore, to synthesise MoC impact on end-user outcomes, we used quality of healthcare domains [17]. This review highlighted inconsistencies with outcome reporting. Within the rural and remote and child health context, the complicated challenges faced by communities, services, and children and their families have necessitated MoCs’ attempt to address multiple complexities. This has in turn resulted in challenges in identifying the impact complex MoCs have on end-user outcomes. Quality was reported in a variety of ways, with no two citations reporting using the same outcome measures. This heterogeneity made generalizability of results difficult. The domain of safety was not reported in the outcomes, which may be due to the nature of the review topic as AHPs seldom engage with modalities that have the potential to cause serious harm or death. Also, it could also be considered that safety is a culmination of all other domains combined (i.e., a service that is effective, efficient, equitable, timely, and patient-centred could be considered as safe). Not surprisingly, effectiveness would be the most frequently identified domain of quality. Across all domains reported on, positive outcomes were generally observed, although this was not universally shared across all the included citations. A gap identified within the literature was a lack of the inclusion of children in evaluating their own healthcare. Patient-centred outcomes focused on parents with only one citation including children in their evaluation. Therefore, due to these complexities, it is unclear if one MoC is superior to another.

### 4.3. Strengths and Limitations

This review has several strengths. Firstly, rigorous and transparent methods were used, with guidelines for best practice for conducting and reporting scoping reviews followed. Secondly, the protocol, extraction, and synthesis of this review was produced by all members of the research team with expertise in not only research methodology but also a deep understanding of the content. In addition to this, an academic librarian was utilised in the development of an appropriate search strategy to encapsulate all required terms. The search was not limited to language to allow for a range of developed countries to be included. Finally, the inclusion of academic and grey literature, as well as quantitative and qualitative data, has added depth to the findings. However, despite these strengths, there are limitations. Quality appraisal of included papers is not a requirement of scoping reviews; however, there were low levels of evidence included in this review. Only two randomised–controlled trials and one pilot randomised–controlled trial were included. Methodologically robust studies are required when evaluating effectiveness of various MoCs. Such research will help to investigate important building blocks for impactful MoCs. However, for translating evidence to practice, where randomised–controlled trials have limited value, we can draw upon the field of implementation science where the adaptation and customisation of evidence into practice can be evaluated in real-world settings. Searching for this review was complex due to broad definitions for each concept (child, AHP, child development, rural, and service delivery). This coupled with heterogeneity in the measurement of outcomes makes it difficult to establish generalizability of the findings. Finally, given this review focused on developed countries, future research could explore what MoCs have been trialled in developing countries. 

## 5. Conclusions

There continues to be challenges when delivering child and family development allied health services in rural and remote areas. As a result, a multitude of MoCs have been developed and implemented. Our findings indicate that most MoCs used in these areas involve role substitution, online-based services, consultative services, or screening services. Irrespective of MoCs, positive outcomes were reported across five of the six domains of quality. While these are encouraging findings, further research is required to strengthen the evidence base. These include understanding which components of MoCs contribute to the greatest positive impact, robust data to measure these impacts, and involvement of end-users in the MoC creation. 

## Figures and Tables

**Table 1:** Summary of inclusion and exclusion criteria for scoping review.

	Inclusion	Exclusion
Population	Children aged ≤18 years Children with conditions related to their developmental health (physical, social, emotional, behavioural, or learning), including diagnosed disability [29] Parents/carers of children with developmental needs Allied health professionals (AHPs) for children with developmental needs Allied health assistants working with supervision from an AHP Students of included allied health professions	Adults (people > 18) receiving the intervention Children with organic disease as the primary diagnosis [29] Weight management services Lifestyle interventions for non-developmental concerns (i.e., diet programs, physical activity programs) Mental illness with no development relation
Concept	Allied health models of care	Acute medical paediatric services Complementary and Alternative Medicines Validity, reliability, or feasibility citations of screening tools Breastfeeding when support is only provided by nurses or medical staff Citations not including the delivery of a health service
Context	Rural and remote areas within developed countries * [26,27]	Papers with mixed participants where data specific to children, or rural/remote areas, could not be extracted. Outcomes that did not relate to or impact the child’s health (e.g., measured AHP perspectives)

* Rural and remote areas in Australia were defined by the Modified Monash Model (MMM) [25]. MMM 2–5 were included within this paper. Rural areas outside of Australia were based on labelling of rurality within the citation. Developed countries were included as per previous research [26] and as defined by the United Nations [27].

**Table 3:** Identified Models of Care.

Study	Screening	Consultative	Role Substitution	Online Services
				
Australian Institute of Health and Welfare 2021 [38]	●			
Autism Spectrum Australia 2021 [39]			●	●
Bohlen, G. 1996 [52]	●			
Chase et al., 2008 [42]		●		
Davies, S. 2007 [36]			●	
Dettwiller and Brown 2015 [32]	●		●	
Dodd et al., 2019 [43]			●	
Fairweather et al., 2016 [53]	●			●
Heins, K. 1998 [44]			●	
Hines et al., 2019 [45]				●
Hoffman et al., 2019 [46]		●	●	●
Hsieh et al., 2020 [55]		●	●	●
Jessiman, S. 2003 [47]			●	●
Johnsson et al., 2018 [40]		●	●	●
Jones et al., 2015 [31]	●		●	
Kirby et al., 2018 [33]	●		●	
Langbecker et al., 2019 [34]				●
Lim et al., 2020 [48]		●		●
Mathisen et al., 2016 [49]	●			
Nevada Department of Human Services 1997 [50]	●			●
Royal Far West 2022 [37]	●		●	
Short et al., 2016 [51]				●
Swift et al., 2009 [54]		●		●
Turner-Brown et al., 2016 [35]		●	●	
Williams and Healy 2007 [41]	●			

**Table 4:** Quality Outcomes.

Study	Timely 1—Referral Pathways	Timely 2—Increasing Availability	Timely 3—Reducing Waiting Lists	Effective 1—Screening and Diagnosis	Effective 2—Benefit of Service	Equitable 1—Accessibility	Equitable 2—Capacity of AHPs	Equitable 3—High Standard	Patient-Centred 1—Health Literacy	Patient-Centred 2—Including Families	Patient-Centred 3—Co-Creation and Co-Design	Efficient 1—Use of Resources
												
**AIHW [38]**	↑ (+)			↕ (+/−)	↕ (+)				↑ (+)			
**ASPECT [39]**					↑ (+)	↑ (+)	↑ (+)			↑ (+)		
**Bohlen, G. [52]**	↕ (+)			↑ (+)								
**Chase et al. [42]**	↑ (+)								↔			
**Davies, S. [36]**		↑ (+)				↑ (+)			↑ (+)			
**Dettwiller and Brown [32]**					↑ (+)			↑ (+)				
Dodd et al. [43]		↓ (+)			↑ (+)	↑ (+)						
**Fairweather et al. [53]**					↑ (+)	↔				↔	↑ (+)	
**Heins, K. [44]**		? (+)	↓ (+)		↑ (+)	↑ (+)						
**Hines et al. [45]**					↑ (+)	↔				↔	? (+)	
**Hoffman et al. [46]**					↑ (+)	↑ (+)		↑ (+)			↑ (+)	
**Hsieh et al. [55]**					↔ *				↔	↑ (+)	? (+)	
**Jessiman, S. [47]**					↑ (+)	↓ (+)				↑ (+)		↓ (+)
**Johnsson et al. [40]**					↔	↑ (+)	↑ (+)			↔		
**Jones et al. [31]**	↑ (+)			↑ (+)		↑ (+)						
**Kirby et al. [33]**	? (+)	↑ (+)			↕ (+/−)					↑ (+)		
**Langbecker et al. [34]**					↑ (+) *	↑ (+)						
**Lim et al. [48]**					↕ (+/-)			↔			↔	
**Mathisen et al. [49]**								↑ (+)	↔	↑ (+)		
**NDHS [50]**					? (+)			↑ (+)		↑ (+)		↑ (+)
**Royal Far West [37]**	↑ (+)			↑ (+)		↑ (+)				↑ (+)		
**Short et al. [51]**		↓ (+)			↑ (+)		? (+)					↑ (+)
**Swift et al. [54]**			↕ (+)		↑ (+) *					↑ (+)		
**Turner-Brown et al. [35]**					↔ *	↑ (+)					↔	
**Williams and Healy [41]**	↑ (+)			↕ (+)			↓ (+)		↑ (+)		↑ (+)	

AHPs—Allied Health Professionals; AIHW—Australian Institute of Health and Welfare; ASPECT—Autism Spectrum Australia; NDHS—Nevada Department of Human Services; ↑ = upward trend; ↓ = downward trend; ↕ = mixed upward and downward trend; ↔ = no change/improvement; ? = trend unclear; (+) = positive change or improvement; (−) = negative change or improvement; * = statistically significant results (*p* < 0.05).

## Data Availability

The original contributions presented in the study are included in the article/Supplementary Material; further inquiries can be directed to the corresponding author/s.

## Supplementary Materials

The following supporting information can be downloaded at: https://www.mdpi.com/article/10.3390/ijerph21040507/s1, File S1: Medline Search Strategy; File S2: Quality domain definitions and subdomains.

## References

[1] Australian Institute of Health and Welfare AIHW Rural and Remote Health. https://www.aihw.gov.au/reports/rural-remote-australians/rural-and-remote-health.

[2] National Rural Health Alliance NRHA (2023). Rural Health in Australia Snapshot 2023.

[3] Smith K.B., Humphreys J.S., Wilson M.G. (2008). Addressing the health disadvantage of rural populations: How does epidemiological evidence inform rural health policies and research?. Aust. J. Rural Health.

[4] Wakerman J., Humphreys J.S., Wells R., Kuipers P., Entwistle P., Jones J. (2008). Primary health care delivery models in rural and remote Australia–a systematic review. BMC Health Serv. Res..

[5] Gallego G., Dew A., Lincoln M., Bundy A., Chedid R.J., Bulkeley K., Brentnall J., Veitch C. (2017). Access to therapy services for people with disability in rural A ustralia: A carers’ perspective. Health Soc. Care Community.

[6] Blayden C., Hughes S., Nicol J., Sims S., Hubbard I.J. (2017). Using secondments in tertiary health facilities to build paediatric expertise in allied health professionals working in rural New South Wales. Aust. J. Rural Health.

[7] (2022). Department for Education Skills and Employment. *Australian Early Development Census National Report 2021: Early Childhood Development in Australia*; Canberra, Australia. https://www.aedc.gov.au/resources/detail/2021-aedc-national-report.

[8] Smith J.D. (2016). Australia’s Rural, Remote and Indigenous Health.

[9] Shonkoff J.P. (2003). From Neurons to Neighborhoods: Old and New Challenges for Developmental and Behavioral Pediatrics. J. Dev. Behav. Pediatr..

[10] World Health Organization (2023). Nurturing Care Framework Progress Report 2018–2023: Reflections and Looking Forward.

[11] Bell A., Corfield M., Davies J., Richardson N. (2010). Collaborative transdisciplinary intervention in early years putting theory into practice. Child Care Health Dev..

[12] Majnemer A. (1998). Benefits of early intervention for children with developmental disabilities. Semin. Pediatr. Neurol..

[13] SARRAH (2016). Models of Allied Health Care in Rural and Remote Australia.

[14] Nancarrow S.A., Roots A., Grace S., Moran A.M., Vanniekerk-Lyons K. (2013). Implementing large-scale workforce change: Learning from 55 pilot sites of allied health workforce redesign in Queensland, Australia. Hum. Resour. Health.

[15] Nancarrow S., Moran A., Boyce R. (2012). Evaluation of a system of monitoring allied health service provision, quality and outcomes. GSTF J. BioSciences.

[16] Bradford N.K., Caffery L.J., Smith A.C. (2016). Telehealth services in rural and remote Australia: A systematic review of models of care and factors influencing success and sustainability. Rural Remote Health.

[17] Committee on Quality of Health Care in America (2001). Crossing the Quality Chasm: A New Health System for the 21st Century.

[18] Kruk M.E., Gage A.D., Arsenault C., Jordan K., Leslie H.H., Roder-DeWan S., Adeyi O., Barker P., Daelmans B., Doubova S.V. (2018). High-quality health systems in the Sustainable Development Goals era: Time for a revolution. Lancet Glob. Health.

[19] Dougherty D., Chen X., Gray D.T., Simon A.E. (2014). Child and Adolescent Health Care Quality and Disparities: Are We Making Progress?. Acad. Pediatr..

[20] Farr J., Moore A., Bruffell H., Hayes J., Rae J.P., Cooper M. (2021). The impact of a needs-based model of care on accessibility and quality of care within children’s mental health services: A qualitative investigation of the UK i-THRIVE Programme. Child Care Health Dev..

[21] Aultman J., Bassi M., Richner G., Delahanty S., Rush S., Spalding S., Grossoehme D. (2023). Fulfilling 6 Domains of Health Care Quality: A Qualitative Parental Caregiver Study of Pediatric Telehealth During a Pandemic. Clin. Pediatr..

[22] Tricco A.C., Lillie E., Zarin W., O’Brien K.K., Colquhoun H., Levac D., Moher D., Peters M.D., Horsley T., Weeks L. (2018). PRISMA extension for scoping reviews (PRISMA-ScR): Checklist and explanation. Ann. Intern. Med..

[23] Canadian Agency for Drugs and Technologies in Health CADTH CADTH Grey Matters. https://greymatters.cadth.ca/.

[24] Turnbull C., Law D., Ashworth E., Grimmer-Somers K., Kumar S., May E. (2009). Allied, Scientific and Complementary Health Professionals: A New Model for Australian Allied Health. Aust Health Rev.

[25] Department of Health and Aged Care Modified Monash Model. https://www.health.gov.au/topics/rural-health-workforce/classifications/mmm.

[26] Moran A., Nancarrow S., Cosgrave C., Griffith A., Memery R. (2020). What works, why and how? A scoping review and logic model of rural clinical placements for allied health students. BMC Health Serv. Res..

[27] United Nations (2019). World Economic Situations and Prospects Country Classification.

[28] Zurynski Y., Phu A., Deverell M. (2015). Paediatric Services Capacity: An Evidence Check Rapid Review Brokered by the Sax Institute for the NSW Kids and Families.

[29] Arefadib N., Moore T. (2017). Reporting the Health and Development of Children in Rural and Remote Australia.

[30] Ritchie J., Spencer L. (2002). Qualitative Data Analysis for Applied Policy Research. The Qualitative Researcher’s Companion.

[31] Jones D., Lyle D., Brunero C., McAllister L., Webb T., Riley S. (2015). Improving health and education outcomes for children in remote communities: A cross-sector and developmental evaluation approach. Gatew. Int. J. Community Res. Engagem..

[32] Dettwiller P., Maroney T., Brown L. Speaking Easy for Living and Learning: School-based service-learning for speech pathology students. Proceedings of the 13th National Rural Health Conference.

[33] Kirby S., Lyle D., Jones D., Brunero C., Purcell A., Dettwiller P. (2018). Design and delivery of an innovative speech pathology service-learning program for primary school children in Far West NSW, Australia. Public Health Res. Pract..

[34] Langbecker D.H., Caffery L., Taylor M., Theodoros D., Smith A.C. (2019). Impact of school-based allied health therapy via telehealth on children’s speech and language, class participation and educational outcomes. J. Telemed. Telecare.

[35] Turner-Brown L., Hume K., Boyd B.A., Kainz K. (2019). Preliminary efficacy of Family Implemented TEACCH for Toddlers: Effects on parents and their Toddlers with Autism Spectrum Disorder. J. Autism Dev. Disord..

[36] Davies S. (2007). The Rural Beginnings Project: A pilot project for early childhood intervention. Team Around The Child.

[37] Royal Far West (2022). Early Learning, Intervention and Screening in Rural and Remote Areas.

[38] Australian Institute of Health and Welfare (2021). Queensland’s Deadly Ears Program: Indigenous Children Receiving Services for Ear Disease and Hearing Loss 2007–2019.

[39] Autism Spectrum Australia (2021). Therapy Assistant Rural Workforce Development—Research Report.

[40] Johnsson G.K.R., Crook S., Cribb C., Rodrigues C. (2018). Online Professional Development and Support in Rural and Remote Disability Services: Innovative Workforce Fund Evaluation and Learning Plan.

[41] Williams J., Healy S. (2007). Busy Bee Screenings: Early Childhood Intervention in a Rural Setting.

[42] Chase P.A., Boggs T.L. (2008). Speech-language-hearing health disparities in young children. Perspect. Sch. Based Issues.

[43] Dodd B., Castles J., Aar M., Hally V., McKimmie J., Mitchell N., Tibbetts S., Wong M., Keage M. (2019). A rural clinical placement: Children’s outcomes. J. Clin. Pract. Speech-Lang. Pathol..

[44] Heins K. (1998). Collaborating for language, speech and literacy—A practical model of a school-based speech pathology service in rural Australia. Aust. J. Early Child..

[45] Hines M., Bulkeley K., Dudley S., Cameron S., Lincoln M. (2019). Delivering Quality Allied Health Services to Children with Complex Disability via Telepractice: Lessons Learned from Four Case Studies. J. Dev. Phys. Disabil..

[46] Hoffmann A.N., Bogoev B.K., Sellers T.P. (2019). Using Telehealth and Expert Coaching to Support Early Childhood Special Education Parent-Implemented Assessment and Intervention Procedures. Rural Spec. Educ. Q..

[47] Jessiman S.M. (2003). Speech and languages services using telehealth technology in remote and underserviced areas. J. Speech-Lang. Pathol. Audiol..

[48] Lim J.M., McCabe P., Purcell A. (2020). Look at Mummy: Challenges in training parents to deliver a home treatment program for childhood apraxia of speech in a rural Canadian community. Rural Remote Health.

[49] Mathisen B., Bennett S., Lockett C., Beazley K., Howlett J., Charlesworth M., Lees H., Read J. (2016). Talking Matters Bendigo: Engaging Parents Early to Prevent Long-Term Speech, Language and Communication Needs in Preschool-Aged Children. Child. Aust..

[50] Nevada Department of Human Services (1997). HAPPY Rural Outreach Project. Final Report.

[51] Short L., Rea T., Houston B., Scott S., Forducey P. (2016). Positive Outcomes for Speech Telepractice as Evidence for Reimbursement Policy Change. Perspect. ASHA Spec. Interest Groups.

[52] Bohlen G. (1996). The early intervention team--a model for multi-institutional cooperation in diagnosis of child developmental delay in a rural district. Prax. Der Kinderpsychol. Kinderpsychiatr..

[53] Fairweather G.C., Lincoln M.A., Ramsden R. (2016). Speech-language pathology teletherapy in rural and remote educational settings: Decreasing service inequities. Int. J. Speech-Lang. Pathol..

[54] Swift M.C., Roeger L., Walmsley C., Howard S., Furber G., Allison S. (2009). Rural children referred for conduct problems: Evaluation of a collaborative program. Aust. J. Prim. Health.

[55] Hsieh Y.-H., Liao H.-F., Jeng S.-F., Tseng M.-H., Schiariti V., Tsai M.-Y., Sun S.-C. (2020). Collaborative Home-Visit Program for Young Children With Motor Delays in Rural Taiwan: A Pilot Randomized Controlled Trial. Phys. Ther..

[56] Clanchy K.M., Baque E., Headrick J., Irvine-Brown L., Sulek R. (2022). Thinking beyond impairment: Recommendations from contemporary models of care for working with children and disability. Aust. J. Gen. Pract..

[57] Dew A., Bulkeley K., Veitch C., Bundy A., Lincoln M., Glenn H., Gallego G., Brentnall J. (2014). Local therapy facilitators working with children with developmental delay in rural and remote areas of western New South Wales, Australia: The ‘Outback’ service delivery model. Aust. J. Soc. Issues.

[58] Mingebach T., Kamp-Becker I., Christiansen H., Weber L. (2018). Meta-meta-analysis on the effectiveness of parent-based interventions for the treatment of child externalizing behavior problems. PLoS ONE.

[59] Mutsekwa R.N., Wright C., Byrnes J.M., Canavan R., Angus R.L., Spencer A., Campbell K.L. (2022). Measuring performance of professional role substitution models of care against traditional medical care in healthcare—A systematic review. J. Eval. Clin. Pract..

[60] Van Hoof S.J.M., Quanjel T.C.C., Kroese M.E.A.L., Spreeuwenberg M.D., Ruwaard D. (2019). Substitution of outpatient hospital care with specialist care in the primary care setting: A systematic review on quality of care, health and costs. PLoS ONE.

[61] Marks D., Comans T., Bisset L., Scuffham P.A. (2017). Substitution of doctors with physiotherapists in the management of common musculoskeletal disorders: A systematic review. Physiotherapy.

[62] Bu S., Smith A.B., Janssen A., Donnelly C., Dadich A., Mackenzie L.J., Smith A.L., Young A.L., Wu V.S., Smith S.J. (2022). Optimising implementation of telehealth in oncology: A systematic review examining barriers and enablers using the RE-AIM planning and evaluation framework. Crit. Rev. Oncol. Hematol..

[63] Sotzen J.R., Stratman E.J. (2023). Geographic variability in rural patient internet connectivity when accessing telehealth services from home: A retrospective analysis during the COVID—19 pandemic. J. Rural Health.

[64] Champion V.L., Monahan P.O., Stump T.E., Biederman E.B., Vachon E., Katz M.L., Rawl S.M., Baltic R.D., Kettler C.D., Zaborski N.L. (2022). The Effect of Two Interventions to Increase Breast Cancer Screening in Rural Women. Cancers.

[65] Leese G.P., Ahmed S., Newton R.W., Jung R.T., Ellingford A., Baines P., Roxburgh S., Coleiro J. (1993). Use of mobile screening unit for diabetic retinopathy in rural and urban areas. BMJ.

[66] Connolly M., Gersch I. (2013). A support group for parents of children on a waiting list for an assessment for autism spectrum disorder. Educ. Psychol. Pract..

