# Supplementary material for: Exploring Allied Health Models of Care for Children with Developmental Health Concerns, Delays, and Disabilities in Rural and Remote Areas: A Systematic Scoping Review
Source: Int J Environ Res Public Health. 2024 Apr 19;21(4):507. doi: 10.3390/ijerph21040507 (PMC11050593; doi:10.3390/ijerph21040507)
Supplement: Supplementary file 1 [file ijerph-21-00507-s001.zip › Supplementary File S1_MEDLINESearchSyntax.pdf]

Supplementary File S1: MEDLINE SEARCH SYNTAX

1. exp Child/
2. exp Infant/
3. Adolescent/
4. Pediatrics/
5. Child Health/
6. Disabled Children/
7. (child* or preschool* or pre school* or school age* or schoolage* or schoolchild* or pediatric* or paediatric* or peadiatric* or infant* or newborn* or neonate* or baby or babies or boy or boys or girl or girls or youth or adolescen* or teen* or juvenil* or prepubsecen* or pubescent* or preteen* or kid or kids or puber* or toddler*).ti,ab,kf.
8. or/1-7
9. Allied Health Personnel/
10. Community Health Services/
11. Community Health Workers/
12. Physical Therapist Assistants/
13. Speech-Language Pathology/
14. Allied Health Occupations/
15. Occupational Therapy/
16. Occupational Therapists/
17. Physical Therapy Specialty/
18. Physical Therapy Modalities/
19. Physical Therapists/
20. Social Workers/
21. Social Work/
22. Dietetics/
23. Nutritionists/
24. Podiatry/
25. Early Intervention, Educational/
26. Psychology/ and (therap* or service* or clinic* or intervention* or program* or appointment*).ti,ab,kf.
27. (allied health or community health or physical therap* or physiotherap* or audiolog* or ((Speech or Language) adj3 (therap* or patholog*)) or occupational therap* or social service* or social intervention* or social work* or dietetics or dietician* or nutritionist* or psychologist* or (psycholog* and (therap* or service* or clinic* or intervention* or program* or appointment*)) or podiatr* or chiropod* or early intervention* or head start program*).ti,ab,kf.
28. or/9-27
29. 8 and 28
30. Child Health Services/
31. 29 or 30
32. Developmental Disabilities/
33. exp Child Development/
34. Child Behavior Disorders/
35. Intellectual Disability/
36. Language Development Disorders/
37. neurodevelopmental disorders/
38. motor skills disorders/
39. Learning Disabilities/

40. ((development* or neurodevelopment* or motor or intellectual or language or speech or psychological or physical or learning or behavio*) adj3 (delay* or vulnerab* or problem* or disab* or disorder* or deviat*)).ti,ab,kf.
41. ((child* or infant*) adj3 development*).ti,ab,kf.
42. or/32-41
43. Rural Health/
44. Rural Health Services/
45. Rural Population/
46. Hospitals, Rural/
47. (rural* or regional or remote* or non urban or non metro* or nonurban or nonmetro* or geographic* isolat* or farming communit* or agricultural communit* or small town*).ti,ab,kf.
48. or/43-47
49. 31 and 42 and 48
