# Supplementary material for: Exploring Allied Health Models of Care for Children with Developmental Health Concerns, Delays, and Disabilities in Rural and Remote Areas: A Systematic Scoping Review
Source: Int J Environ Res Public Health. 2024 Apr 19;21(4):507. doi: 10.3390/ijerph21040507 (PMC11050593; doi:10.3390/ijerph21040507)
Supplement: Supplementary file 1 [file ijerph-21-00507-s001.zip › Supplementary File S2_QualityDomains.pdf]

Supplementary File S2 – Quality domains definitions and subdomains

Effective: *Providing services based on scientific knowledge to all who could benefit and refraining from providing services to those not likely to benefit (avoiding underuse and misuse, respectively)*
Effective 1: Improving screening, assessment and diagnostic processes to ensure services are delivered to those who are likely to benefit and not providing services to those not likely to benefit.
Effective 2: Evaluating the benefit of a service.

Efficient: *avoiding waste, including waste of equipment, supplies, ideas, and energy.*
Efficient 1: Optimising the use of resources (human, financial and infrastructure) to avoid waste.

Equitable: *providing care that does not vary in quality because of personal characteristics such as gender, ethnicity, geographic location, and socioeconomic stats.*
Equitable 1: Improving accessibility of services (i.e., reducing travel times, improving presence of services in areas).
Equitable 2: Increasing capacity of AHPs in rural areas. (To ensure the workload of rural practitioners does not vary from those in urban areas).
Equitable 3: Ensuring a high standard and reducing the variability of healthcare irrespective of the location.

Timeliness: *Reducing waits and sometimes harmful delays for both those who receive and those who give care.*
Timeliness 1: Improving referral pathways to ensure people receive appropriate care – increasing access to receiving a referral and decreasing unnecessary referrals.
Timeliness 2: Increasing availability of professionals to reduce delays of those who give care.
Timeliness 3: Reducing waiting lists to prevent harmful delays.

Patient-centredness: *providing care that is respectful of and responsive to individual patient preferences, needs, and values and ensuring that patient values guide all clinical decisions. For the purpose of this paper, the "patient" was the child and their parent/caregiver. This was decided due to the nature of paediatric care.*
Patient-Centred 1: Improving health literacy so that patients are able to appropriately advocate for and receive healthcare that aligns with their needs and values.
Patient-Centred 2: Including family and child perspectives of the service to ensure patient values are guiding clinical decisions.
Patient-Centred 3: Co-designing and co-delivering services with families so they are responsive to the family's needs
